# Archaeal and Extremophilic Bacteria from Different Archaeological Excavation Sites

**DOI:** 10.3390/ijms24065519

**Published:** 2023-03-14

**Authors:** J. Michael Köhler, Linda Ehrhardt, P. Mike Günther

**Affiliations:** Department of Physical Chemistry and Microreaction Technology, Institute for Micro- und Nanotechnologies/Institute for Chemistry and Biotechnology, Technische Universität Ilmenau, 98684 Ilmenau, Germany; linda.ehrhardt@tu-ilmenau.de (L.E.);

**Keywords:** soil, bacterial communities, Archaea, NGS, archaeology, tannery, mining, copper, saline

## Abstract

Beside natural factors, human activities are important for the development of microbiomes. Thus, local soil bacterial communities are affected by recent activities such as agriculture, mining and industry. In addition, ancient human impacts dating back centuries or millennia have changed soils and can emboss the recent bacterial communities up to now, representing a certain long-term “memory of soil”. Soil samples from five different archaeological excavation places were investigated for the presence of *Archaea* with a Next Generation Sequencing (NGS) analysis of the DNA coding for 16S r-RNA sequences. It was found that the abundance of *Archaea* differs strongly between less than one and more than 40 percent of bacteria. A Principal Component Analysis (PCA) of all samples shows that the archaeological excavation places can be distinguished from each other by the archaeal component of soil bacterial communities, which presents a typical pattern for each place. Most samples are marked by the dominance of *Crenarchaeota*, which are presented mainly by ammonia-related types. High contents of *Nanoarchaeaota* have been observed in one ash deposit of a historical saline and all samples of a historical tannery area. These samples are also marked by a significant presence of *Dadabacteria*. The specific abundancies of special Archaea—among them ammonia-oxidizing and sulphur-related types—are due obviously to former human activities and support the concept of the “ecological memory of soil”.

## 1. Introduction

The character of the soil, its fertility and robustness are closely connected with the composition of soil bacterial communities [1]. Soils not only contain a huge number of bacteria, but represent, in general, ecosystems of very high diversity. Typically, a few bacterial types are present in high cell numbers, whereas a vast number of different bacteria form a microbial background in a more or less dormant state [2]. Therefore, most soils have an extensive reservoir of very different genetic resources, which can be activated when the environmental conditions are changed [3]. This community background is the basis for the robustness of soil microbial communities. This allows them to adapt to the slow drifts in the character of an environment as well as in the case of immediate drastic changes.

Each volume element of soil changes the quantitative and qualitative compositions of its bacterial community due to the shifts and breaks during the evolution of the environment, including macro flora and fauna as well as geological and other physical processes and the accompanying chemical changes. Bacteria which had been essential for physiological activity during an earlier phase disappear or might fall into a dormant state under new environmental conditions in a later phase. In this sense, the composition of “silent bacteria” stores ecological information about the past to some extent. The large number of less or non-active soil bacteria forms a kind of “ecological memory” of soil.

In addition to natural factors, human impacts contribute to the development of soil microbial systems. Agriculture, cattle breeding, settlements and handcraft activities lead to changes in the physical and chemical parameters of soil, the exchange of gases, humidity, and so on. In particular, the exploration of natural resources, mining and industrial production has a huge impact on the local environmental conditions and is marked mainly by big changes in soil microbial communities [4,5,6]. A typical, and drastic, example is the development of acid drainage from industrial mining sites [7,8,9].

Since industrialization, the human impact on the environment is very strong and has achieved dangerous global dimensions during the last decades. However, it has to be seen that this impact starts much earlier with the beginning of the formation of culturally influenced habitats—occurring in middle Europe the latest with the introduction of agriculture about 7500 years before. It became enforced with the fabrication of metals and the development of cities. Therefore, not only the modern industrial society but also older cultures have impacted the soils and their bacterial communities [10,11]. 

During the investigation of soil samples from areas with pre-industrial human impact, special bacterial types from the archaeal domain as well as other extremophilic types had been observed several times [12]; for example, *Hadesarchaeota* from the early copper mining places of the East Harz region [13]. *Hadesarchaeota* was first found in deep gold mines in South Africa [14]. This group and other special groups of bacteria such as *Aenigmarchaeota, Asgardarchaea* [15,16] and *Nanoarchaea* [17] have been reported to be detected in extreme environments, in the deep ground, or from deep sea hot spots typically.

Remarkably, a considerable portion of bacterial DNA from near-surface soil indicating such extremophilic types was also found in soil samples from archaeological excavation places. On the one hand, an increasing number of investigations indicate that such bacteria are not exclusively present in the deep ground and the deep sea but are largely distributed. On the other hand, special types of areas used to promote the presence of the above-mentioned archaea. It is to be expected that natural conditions and recent human activities are only one part of the factors for the development of soil bacterial communities. In addition, their composition is modulated by traces of former events and human impacts. Soils and the patterns of their bacterial communities are embossed by their history which includes pre-industrial land use as well as soil-related human activities in the middle age and in prehistorical periods. Therefore, it is assumed that each soil bacterial community includes a certain “memory of soil history”. In addition to other bacteria, even Archaea should be regarded as possible indicators for such a memory effect.

Here, the appearance of such microorganism groups in soil samples from a historical tannery area of the city of Jena and some other archaeological excavation sites is reported as an example of the early human impact on soil microbial communities and their archaeal components.

## 2. Results and Discussion

### 2.1. Presence of Archaeal Phyla in the Archaeological Samples

**Figure 1:**
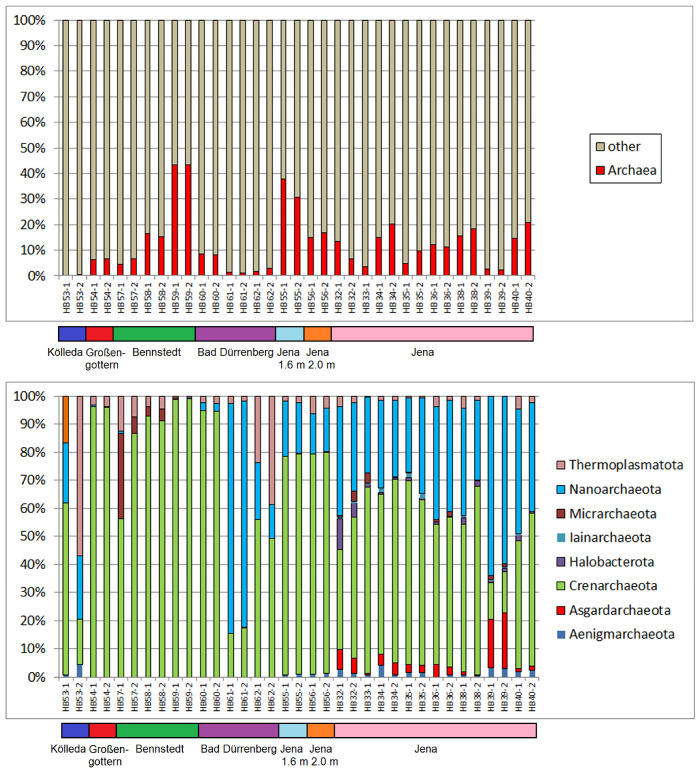
Percentage of Archaea reads in the soil samples: total portion of Archaea.

The regarded soil samples show considerable differences in the presence of Archaea: in most samples between about 5 and 20% (Figure 1). Whereas the samples from Kölleda (HB53-1 and HB53-2) contained less than 1%, the replaced topsoil from the ancient coal mine area of Bennstedt (HB59-1 and HB59-2) is marked by more than 40% of Archaea in the obtained 16S r-RNA reads. More than 30% Archaea content was found in samples HB55-1 and HB55-2 (Jena, Germany, depth 1.6 m). The dominating archaeal phylum in these samples as well as in most of the other samples is *Crenarchaeota*. *Crenarchaeota* are widely abundant in different environments, such as in oceans [18], forests, grasslands, permafrost, and in fresh water, whereby the typical abundance was found to be up to about 3% of bacterial 16S r-RNA genes [19].

The red ash deposit from the saline of Bad Dürrenberg (HB61) contains a much lower portion of Archaea, but in this group *Nanoarchaeota* is the most abundant *Archaea* with about 80% of all *Archaea. Nanoarchaeota* are microorganisms with reduced genomes which are symbionts of other *Archaea* and have been mainly found in marine thermal vents and hot springs [17]. Comparatively high amounts of *Nanoarchaeota* were observed in all samples from the ancient tannery area of the city of Jena. Their high presence in many of the investigated samples suggests the promotion of *Nanoarchaeota* by thermal processes—possibly in connection with application of mineral coal as it was used in the saline processes supplying the ashes of samples HB61-1, -2 and HB62-1, -2.

In addition, most samples from the sample group from the ancient tannery area show a significant presence of *Asgardarchaeota*. This *Archaea* are identified as sharing several features with eukaryotic microorganisms and seem to be one of the closest prokaryotic relatives to the eukaryotes. *Asgardarchaeota* have been found in a wide range of microbiomes on Earth, preferably from sediment habitats [20]. One special aspect of their adaptation to extreme environments is their ability to metabolize hydrocarbons [16]; another aspect is their tolerance against salt stress—probably related to the development of salt-adaptive strategies [15]. The occurrence of *Asgardarcheota* in the soil samples from different sampling sites of the ancient tannery area might be related to the application of sodium chloride and other salts in the tannery and dying processes. Interestingly, *Asgardarchaeota* are present in the upper layers of the tannery area of Jena, from which all samples are marked by comparatively high pH values (see Supplementary Table S2). In contrast, the deeper sediment layers from Jena (HB55, HB56), which are marked by lower electrical conductivity values (lower salt content) and lower organic content, supplied no reads for *Asgardarchaeota.*

In addition to the *Crenarchaeota, Thermoplasmatota* have been proven in nearly all samples. They represent the second highest abundant archaeal phylum in the samples of the grey ash deposit of the Saline of Bad Dürrenberg. *Thermoplasmatota* is one of the most abundant components in archaeal sea plankton [21] and members of this phylum seem to be related to the metabolic oxidation of sulphur compounds [21].

The appearance of *Micrarchaeota* in the samples from the ancient coal mine area near Bennstedt corresponds to the fact that this archaeal phylum is related to acidic environments and can be associated with *Thermoplasmatales* [22]. Indeed, the three samples of Bennstedt showed the lowest pH values of the investigated soil samples (pH between 4.05 and 4.22 [23]).

### 2.2. Principle Component Analysis and Comparison of Samples by Different Archaea Taxons

**Figure 2:**
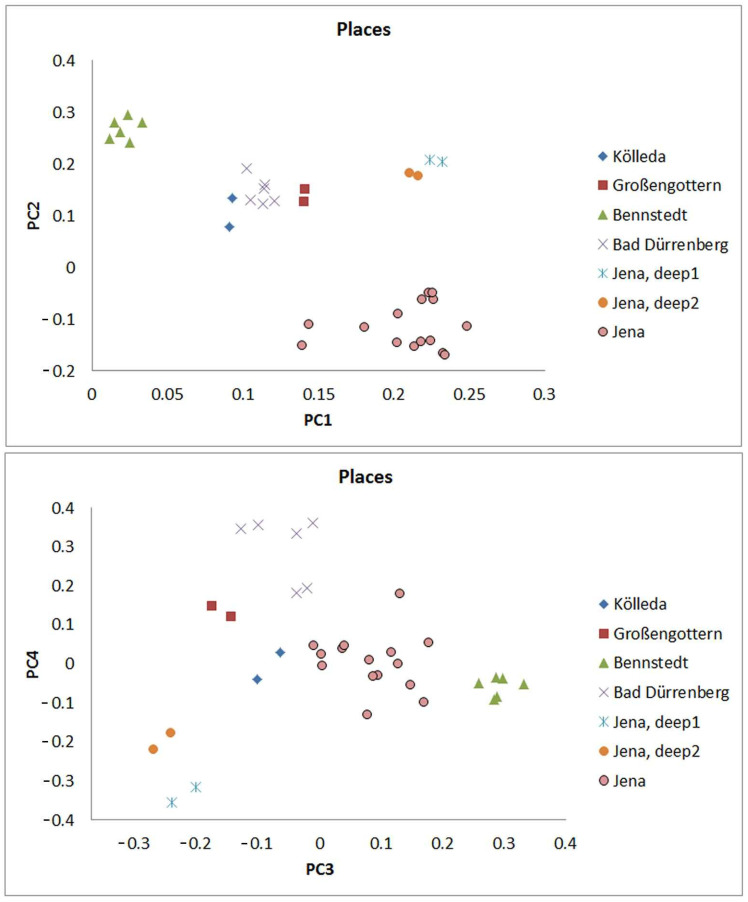
Principal Component Analysis (PCA) by archaeal OTUs; plots for the first, second, third, and fourth principal component (PC1, PC2, PC3 and PC4).

A more detailed comparison of samples can be achieved with a principal component analysis (PCA) on the OTU level. For the calculation, the PCA tool of Mathlab (R2015b) was used. The procedure is based on a minimization of the sum of square deviations of linear distances. The input data are the r-values (logarithmic values calculated following Equation (1)) of the abundances of all found OTUs of the domain of Archaea of all the regarded samples (74 different OTUs in total). These values are applied instead of the absolute read numbers in the PCA in order to include high-abundant and low-abundant types in a balanced way. The results are shown in two-dimensional plots for the first and the second as well as for the third and the fourth principal components (Figure 2).

It is obvious that the different sampling places are clearly distinguished by the PCA plots. Four-point clouds are displayed in the plot for first and second PCA: The central point cloud is formed by the samples from Großengottern, from Bad Dürrenberg and from Kölleda. Well separated from these points and clearly distinct from each other are the samples from Bennstedt, the samples from the deeper layers of Jena (1.60 m and 2 m depth) and the other samples of Jena. The 1.60 m samples and the 2 m sample from Jena are similar in PC3 but separated in PC4 (yellow dots and blue stars in Figure 2). In the PC3/PC4 plot, the samples from Kölleda (blue diamonds), Bad Dürrenberg (pink crosses) and Großengottern (red squares) are separated from each other too (Figure 2).

### 2.3. Specificity of Soil Samples by Abundance of Different Archaea

**Figure 3:**
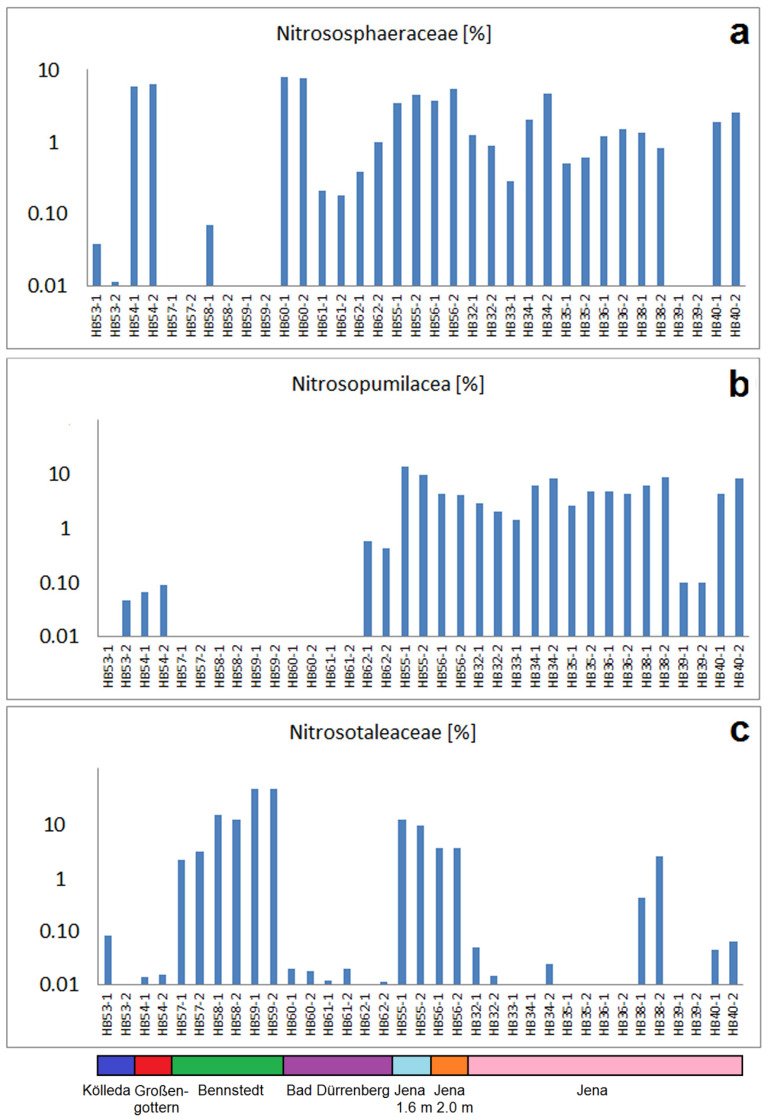
Abundances (percentages of total read numbers) for three families of ammonia-oxidizing *Crenarchaeota* (It has to be reconsidered that percentages below 0.1 are less relevant because they correspond to a few reads only): (**a**) abundances of *Nitrososphaeraceae,* (**b**) abundances of *Nitrosopumilaceae,* (**c**) abundances of *Nitrosotaleaceae*.

Among the highly abundant *Crenarchaeota*, the three families *Nitrososphaeracea, Nitrosopumilaceae* and *Nitrosotalaceae* of the class *Nitrososphaeria (Nitrososphaera)* are the most abundant in the investigated soil samples. *Nitrososphaeria* are known, in general, for their role in nitrogen cycling and are found in heavy metal ion-polluted soils—for example, in mining areas and in acid mine drainage [24,25]. Such mine drainages are typically marked by low pH values, enhanced heavy metal ion concentration—whereby the low pH supports the mobilization and bioavailability of these metal ions—and enhanced salt content. The *Nitrososphaeria* are first isolated from garden soil and recognized for their ability to oxidize ammonia [26]. *Nitrososphaeraceae* are abundant in the majority of the investigated samples (Figure 3a), but particularly high in HB54-1/-2 (Großengottern) and HB60-1/-2 (Bad Dürrenberg- top soil). The abundance in the samples from Großengottern corresponds well with the heavy metal contamination in the local soil, whereas the samples HB60-1/-2 are marked by very high electrical conductivity, indicating the high salt content of the place where the historical ashes had been deposed. In addition, this bacterial group indicates the former release of nitrogen-species as ammonia into the soil, which matches to the former input of nitrogen-riche organic material and waste into the soil. Remarkably, a dominance of *Nitrososphaeraceae* corresponds with the general high abundance of ammonia-oxidizing bacteria in the sample set of Jena. In these soils, a high input of organic material—and in particular animal residues as well as urea—could be expected from the former tannery activities in this area. It is interesting, too, with respect to a possible role of copper, in particular in the case of the soil samples HB54-1 and HB54-2 which were in direct contact with the bronze artefacts of the hoard of Großengottern.

*Nitrosopumilaceae* (Figure 3b) are also highly abundant in the samples from the old tannery area of Jena but are found (above 0.1% of reads) in the sample set of Bad Dürrenberg only in the grey ash deposit (HB62-1 and HB62-2). *Nitrosopumilaceae* are known for their ability to oxidize ammonia [27]. Similar to *Nitrososphaeracea*, they are related to low oxygen availability and are found typically in near-surface sediment layers and in the bathypelagic zone of oceans [28].

The abundance of *Nitrosotaleaceae* shows a rather different distribution picture in the investigated samples (Figure 3c). In the sample set of the tannery area of Jena, they are present in medium or higher read numbers only in samples HB38-1/-2 and in the deep reference samples (HB55-1/-2 and HB56-1/-2). In contrast, they are comparatively highly abundant in the samples of Bennstedt (HB57-1/-2, HB58-1/-2 and HB59-1/-2), which are marked by the absence of *Nitrosopumilaceae.* This characteristic difference between the soil samples of Bennstedt and most other samples is probably due to the low pH of these samples, which corresponds with the described dominance of this family at low pH [29]. However, it has to be remarked that the pH value is probably not the only criterion for the presence of *Nitrosotaleaceae* in the archaeologically taken soils because they are also present in HB38-1/-2, in HB55-1/-2 and HB56-1/-2, which have pH-values around 9.

**Figure 4:**
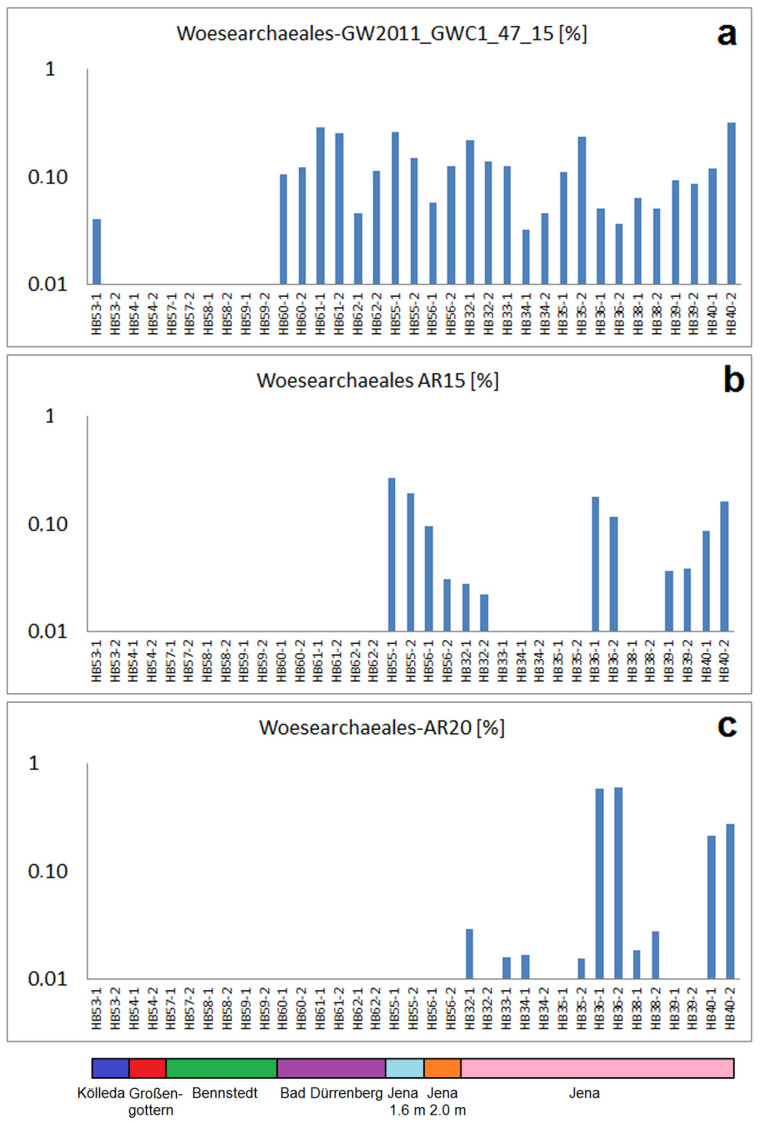
Abundances (percentage of total read numbers) of three OTUs in the order *Woesearchaeales*: (**a**) abundances of the group GW2011_GWC1_47_15, (**b**) abundances of the group *Woesearchaeales* AR15, (**c**) abundances of the group *Woesearchaeales* AR20.

The newly defined phylum *Woesearchaeota* (DPANN superphylum) is represented by three OTUs of the order *Woesearchaeales* (Figure 4). These bacteria have been identified as surprisingly highly abundant in very different habitats, among them oil reservoirs and sulphur springs. Obviously, they are part of a consortia with methanogenic bacteria which are involved in carbon cycling under anaerobic conditions. In addition, they are involved in the nitrogen fixation, denitrification and reduction of sulphate [30,31]. Probably, they are symbionts of methanogenic bacteria in the degradation processes of synthetic polymers in wastewater [32]. The type GW2011_GWC1_47_15 was found in all samples of the tannery area of Jena as well as in the samples from the saline ash deposits of Bad Dürrenberg (Figure 4a). In contrast, the types AR15 and AR20 have been proven in a part of the samples from Jena only, whereby the samples HB36-1/-2 and HB40-1/-2 show particular high abundances for both OTUs (Figure 4b,c).

**Figure 5:**
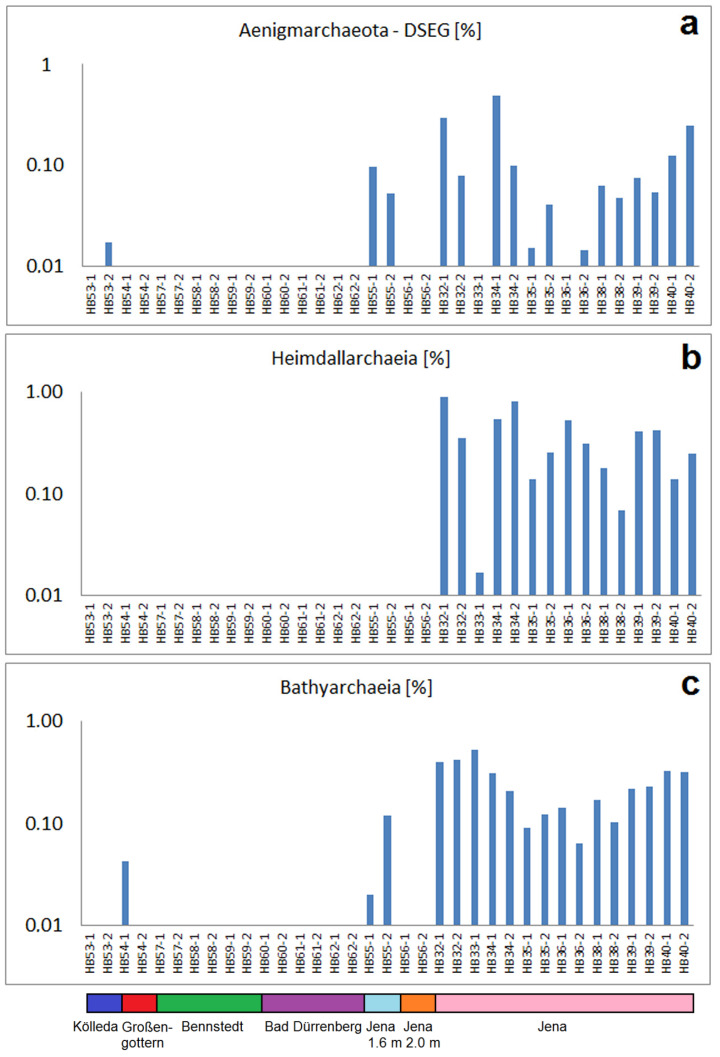
Preferential or exclusive presence of three archaeal groups in the samples from Jena: (**a**) abundances of *Aenigmarchaeota*-DSEG, (**b**) abundances of *Heimdallarchaeia*, (**c**) abundances of *Bathyarchaeaeia*.

The phylum *Aenigmarchaeota* and the classes *Heimdallarchaeia* and *Bathyarchaiea* are mainly found in the samples of Jena too, but not, or very less, in the other samples (Figure 5). The same is the case for some other less abundant archaeal OTUs as *Methanosarcina, Cand. Iainarchaeum, Methanocellales—Rice Cluster* and *Thermoplasmata—DHVEG-1* (Figure S1). *Bathyarchaiea* have been rather recently identified as one of the “most abundant microorganisms on earth” possessing an important spectrum of metabolic abilities including the degradation of proteins, lipids and aromatics such as benzoate [33]. These bacteria are mainly present in anoxic sediments typically characterized by a high abundance of archaeal in the soil bacteria communities [34] In addition to the samples from Großengottern (HB54/1 und HB54/2), they have been found in all samples of Jena, which obviously corresponds to the complex composition of the former input of waste in these soils.

**Figure 6:**
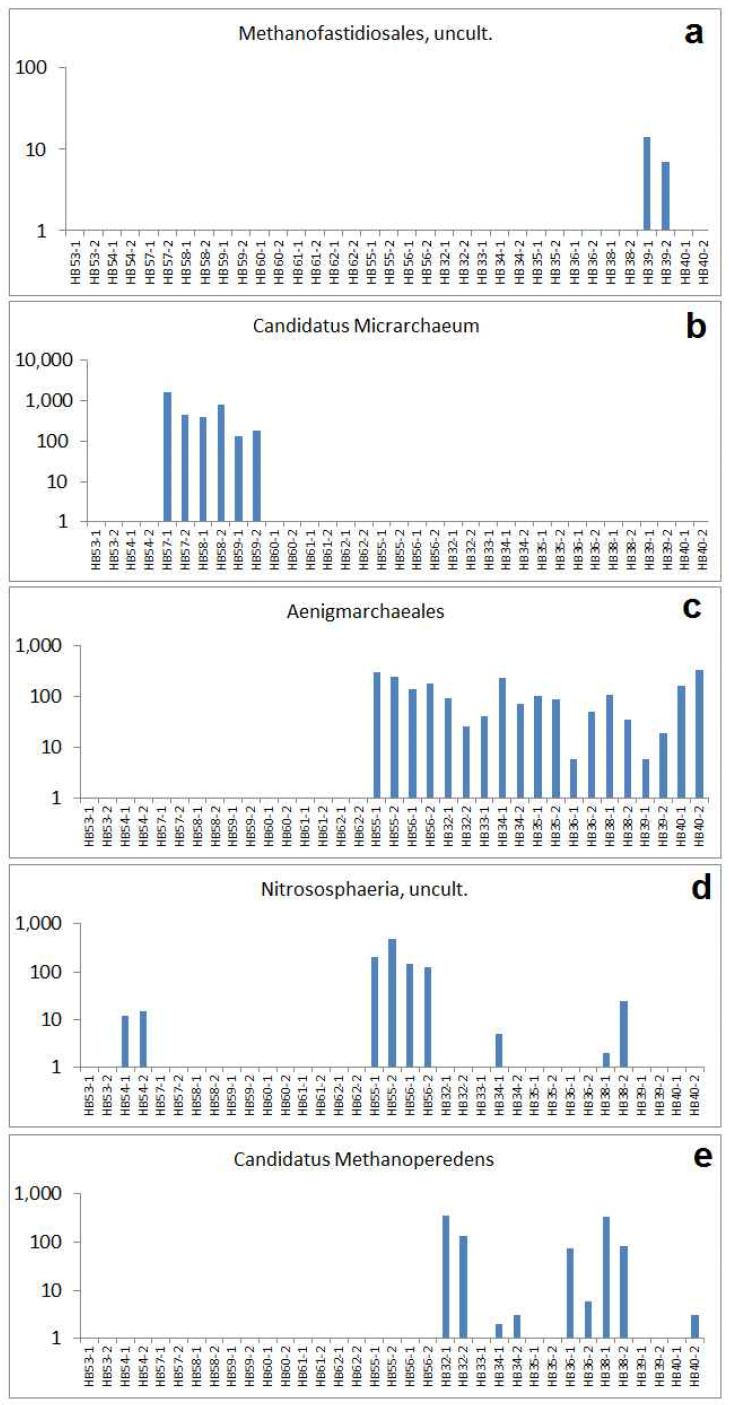
Specific patterns of selected archaeal types reflected by their absolute read numbers: (**a**) abundances of *Methanofastidiosales*, uncult., (**b**) abundances of *Cand. Micrarchaeum*, (**c**) abundances of *Aenigmarchaeales*, (**d**) abundances of *Nitrososphaeria*, uncult., (**e**) abundances of *Cand. Methanoperedens*.

Comparing the different sampling places, certain specificity can be discerned concerning selected OTUs. For example, the euryarchaeotic type *Methanofastidiosales*, uncult. was found in samples HB39-1 and HB39-2 only (Figure 6a), which originated from the interior soil of one vat (Jena). Other OTUs seem to be characteristic for a sample group. This is the case for *Candidatus Micrarchaeum*, which was obtained from the three sampling sites of the early coal mining area of Bennstedt exclusively (Figure 6b). This is caused, obviously, by the general low pH of these soil samples and confirms the acidophilic character of *Micrarchaeum* (see Table S2). In addition, *Nitrososphaeria*, Grp. 1.1c and *Thermoplasmata*, uncult. reflect the special conditions of the samples from Bennstedt. *Aenigmarchaeales* are typical for the samples from the tannery area of Jena. They are found there in all samples, but not in the other sample sets (Figure 6c). The deep-layer reference samples from Jena are marked by a comparatively high number of reads of an OTU named “*Nitrosphaerie, uncult*” (Figure 6d). This OTU was indicated by very few reads in HB34, HB38 (both Jena, outside vat) and additionally in HB54 (Großengottern). Finally, the OTU “*Cand. Methanoperedens*” was found in a part of the samples from Jena only—thereby most abundant in HB32 and HB38 (Figure 6e). This type of Archaea is described as to couple the anaerobic oxidation of methane with the reduction of nitrate [35], which would correspond well with human impact which caused high organic content in the historical and prehistorical soil development of settlement and handcraft areas. This matches to the finding of other nitrate-reducing and methanogenic bacteria in the old tannery area of Jena too. Investigations on sludges from recent tannery activities confirm the importance of *Archaea* in tannery-related soils. The addition of tannery waste in composting supplied a significant shift in the composition of soil bacterial communities [36,37], whereby the relative abundances of *Archaea* have been found higher in anaerobic than in aerobic tannery sludges [38].

### 2.4. Dadabacteria and Zixibacteria

**Figure 7:**
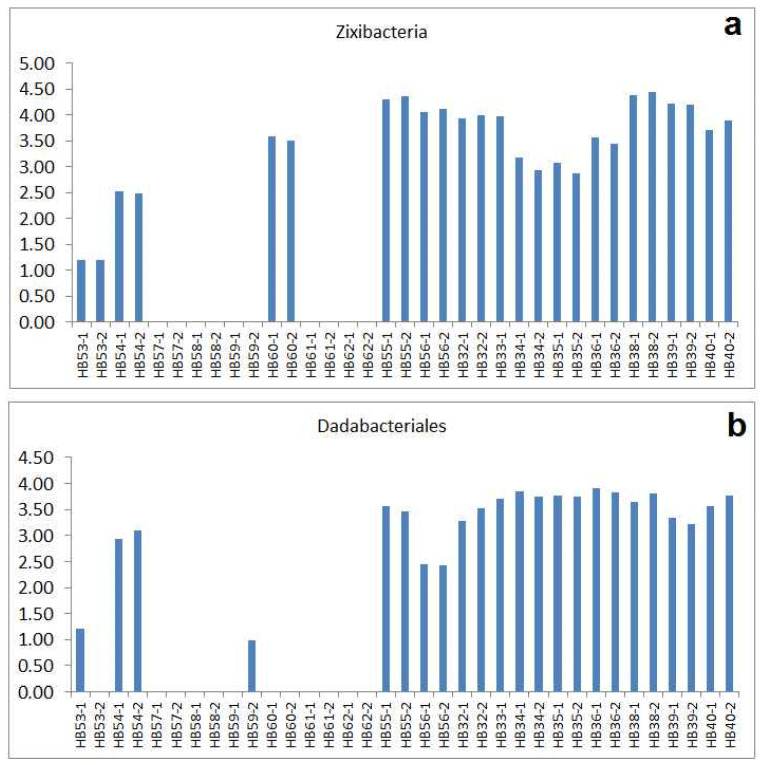
Abundances of *Zixibacteria* and *Dadabacteria* (non-archaeal types) shown by their r-values (normalized logarithmic read numbers corresponding to Equation (1)): (**a**) abundances of *Zixibacteria*, (**b**) abundances of *Dadabacteriales*.

Most samples from the old tannery area of Jena are marked by a remarkably high number of reads for *Dadabacteria* (Figure 7a) and *Zixibacteria* (Figure 7b). Typically, they are represented by between several hundred and several thousand reads. *Dadabacteria* are nearly not found in the samples of Kölleda, Bennstedt and Bad Dürrenberg. A significant number of reads for *Zixibacteria* was observed in HB60-1/-2 (Bad Dürrenberg topsoil) and both types in the samples from Großengottern (HB54-1/-2) only. Recently, *Zixibacteria* has been identified as one of the prevalent phyla in a chemoautotrophical ecosystem driven by hydrogen sulphide-rich groundwater found in a cave in Romania [39]). Sulphur-rich residues from tannery processes such as fur, horn, and hair may have been a source of keratin and sulphur-based metabolic activity in the soil of the former tannery area in Jena.

*Dadabacteria* are known from marine, terrestrial, sub-surface and hydrothermal environments. They preferentially metabolize microbial organic matter and can degrade peptidoglycans and phospholipids in particular. High contents of *Dadabacteria* have been found in coastal sediments too. The highest abundances seem to be correlated with human impact. Thus, *Dadabacteria* are an indicator for pollution and are probably related to the degradation of aromatic compounds [40,41]. In addition to *Archaea* and other bacteria, an enrichment of *Dadabacteria* was reported to be found in recent tannery sludge [42]. This observation can be confirmed by the results reported here which connect the abundance of *Dadabacteria* with the comparatively high abundance of *Archaea* in a historical tannery area.

### 2.5. Discussion of Abundance of Archaea Related to Prehistorical and Historical Burial, Settlement and Workplaces

The composition of soil bacterial communities of archaeological places is of large interest. Despite that fact, up to now few studies on soil bacteria communities of prehistorical and historical settlement and workplaces are available. The results speak for an impact of former human activities which is still reflected in the recent bacterial communities. For example, it is assumed that burial places possess the “highest prokaryotic diversity of any environment” [43], but the knowledge about these bacterial communities is still low [44]. In the soils of the burial sites of Jingzhou, China (about 5th to 3rd century BC), *Crenarchaeota* have been identified, but in a low content next to the more than 95% of *Proteobacteria*, *Actinobacteria*, *Bacteriodetes* and *Firmicutes* ([44], Figure 1). *Archaea* had been found up to a content of about 8% in some soil samples from the ancient copper mining areas of the East Harz region [45]. NGS investigations on soil bacteria communities of historical rice fields speak to the fact that methanogenic Archaea could be used as an indicator for medieval rice cultivation [46].

For comparison with the archaeal components in our investigations, the proof of *Archaea* in soils from a prehistoric settlement of Sicily are particularly interesting: Human impact layers from this iron age settlement (6th century BC) of Monte Iato (Sicily/Italy) showed in the NGS data of archaeal domain a strong dominance of *Nitrososphaera*, a lower content of *Euryarchaeota*, in particular *Methanomassilococcus,* and only low contents of other *Archaea* [47]*. Methanococcus* was also found in low content in one sample from Großengottern, but in several samples from the tannery area of Jena. Different OTUs of the Nitrosospharea have been found in all samples investigated in this presented study. As in the case of the samples from Monte Iato, these ammonia-oxidizing bacteria seem to present a strong indicator of human impact on ancient soil.

## 3. Methods and Materials

### 3.1. Soil Samples

**Figure 8:**
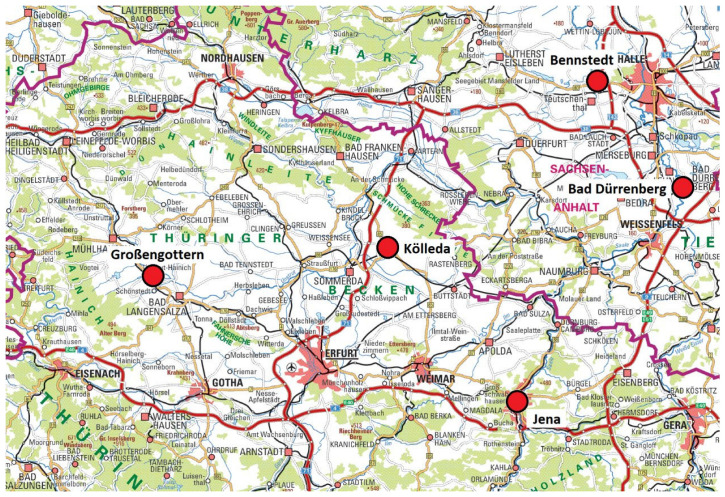
Locations of sampling sites (map: © GDI-Th).

In this study, the results of 16S r-RNA profilings using NGS data from 35 samples from five different archaeological investigated places (Figure 8) were analysed for the presence of selected groups of Archaea. The starting point was the observation that in some of these samples astonishingly high portions of Archaea were present.

All samples were taken during archaeological excavations but under different original motivations. In the case of samples HB53-1 and HB53-2 (Kölleda) and HB54-1 and HB54-2 (Großengottern), soil samples were taken from the direct neighbourhood of bronze artefacts with the original intention to search for heavy metal-tolerant bacterial strains. The samples of Bennstedt and Bad Dürrenberg were taken to compare three sites closely situated to each other with different soil characters: in Bennstedt from a small ancient shaft for a coal mining survey, in Bad Dürrenberg from ash deposits of the local old saline. All other samples originated from a historical tannery area in the city of Jena, where residues of vats of unknown functions had been detected. An overview of included samples is given in Supplementary Table S1.

The samples differ considerably in their chemical properties. General differences in the soil properties of the sampling sites are reflected in the soil pH and the electrical conductivities (Supplementary Table S2). The samples of the ancient coal mining sites of Bennstedt (HB57, HB58 and HB59) are marked by the acidic character of the soil. All other samples show a nearly neutral or moderate alkaline character. The sampling sites of the historical saline in Bad Dürrenberg (HB60, HB61, HB62) are marked by high electrical conductivity values, which can be interpreted as a high salt content of the ash deposits and the related soil.

The investigated soil samples originated from very different archeological situations related to former human activities which led to the expectation of memory effects in soil microbiomes. The samples from Kölleda (HB53-1/-2) and Großengottern (HB54-1/-2) are related to humus soil in immediate contact with deposed pre-historical bronze artefacts. For these samples, impacts could be expected from the temporal release of copper ions and ions of minority alloying metal—depending on changing humidity and pH of near-surface soil—on the one hand, and in general by the buried ancient surface soil, on the other hand. In contrast, the samples from Bennstedt are related to early industrial mining prospection from the time around 1800. Humus material from the historical surface was mainly present in the replaced topsoil (samples HB59-1/-2), whereas the refilling material of the prospection shaft (HB58-1/-2) was low-humus sediment and the material from the coal seam (HB59-1/-2) was first coming to the surface after deposition millions of years ago. All six samples are marked by considerably low pH-values (around 4.1) which speaks to the presence of acidophilic soil microorganisms. The samples from Bad Dürrenberg (HB60-1/-2, HB61-1/-2 and HB62-1/-2) showed higher pH-values (between 7.7 and 8.2) but are marked by high electrical conductivity (above 1 mS/cm), which hints to preferential abundances of halotolerant types. The high conductivity is related to the fact that the soil samples had been taken from ash deposits of a historical saline facility originating from the early industrial period (beginning of the 19th century). The other samples (from Jena, Germany, Inselplatz, HB32-1 to HB40-2) were taken during the excavation of a late medieval to early modern time tannery area. There, human impacts are expected in connection with the deposition of animal waste and feces as well as tannery and dyeing-related materials and chemicals, which speaks to the presence of metal-tolerant as well as of nitrate-, ammonia- and sulphur-compound-related bacteria.

Thus, the investigated soil samples reflect very different archaeological situations and led to the expectation of significant differences in the soil bacterial communities. These differences are analysed in the following using the 16S r-RNA data of the archaeal part of the soil bacterial community.

### 3.2. Sample Processing

DNeasy^®^ PowerSoil^®^ Pro Kits (Qiagen, Hilden, Germany) were applied for the isolation of DNA from soil material following supplier’s instructions. For selective DNA amplification with PCR an Edvocycler (Edvotek, Washington, DC, USA) was used. The quality of PCR products was checked after each PCR step with gel electrophoresis in 1% agarose gels. The primary amplificates as well as the completed pooled libraries were purified following the supplier’s standard protocol with application of the ProNex^®^ Size-Selective Purification System (Promega, Madison, WI, USA).

Adaptor primers Amplicon PCR A519F-Ad (5′ TCGTCGG-CAGCGTCAGATGTGTATAAGAGACAGCAGCMGCCGCGGTAA 3′) and Bact_805R-Ad (5′-GTCTCGTGGGCTCGGAGATGTGTATAAGAGACAGGACTACHVGGGTATCTAATC 3′) were obtained from Eurofins Genomics (Ebersberg, Germany). They were applied in a concentration of 100 pmol/µL. The PCR mixtures (25 µL in total per reaction) were composed as follows: 0.5 µL of DNA isolation eluate, 2 mM MgCl_2_, 200 µM dNTP mix, 0.65 Units GoTaq^®^ G2 Hot Start DNA Polymerase, nuclease-free water (all reagents from Promega, Madison (USA)) and 1 µM of each primer. For PCR amplification, the following steps were performed: initial denaturation for 5 min at 94 °C, 30 amplification cycles involving 30 s denaturation at 94 °C, 30 s primer annealing at 50 °C and 30 s extension at 72 °C. After thermocycling, the process was finished with a final extension reaction at 72 °C for 5 min.

The forward and reverse indexing primers for index PCR were applied in a concentration of 1.25 pmol/µL. They were also obtained with Eurofins Genomics (Ebersberg, Germany). The PCR for the index process was realized using 25 µL per reaction as a total volume composed of 2.5 µL of Amplicon PCR product, 2.5 mM MgCl_2_, 300 µM dNTP mix, 0.5 Units GoTaq^®^ Mdx Hot Start DNA Polymerase, nuclease-free water (all reagents from Promega Corp., Madison, WI 53711 Madison (USA)) and 125 nM of each of the two primers of the respective indexing–primer pairing.

The index–primer PCR involved the following process steps: initial denaturation for 3 min at 95 °C; 30 amplification cycles involving 30 s denaturation at 95 °C; 30 s primer annealing at 55 °C; and 30 s extension at 72 °C. After the 30 cycles of thermocycling, the process was finished with a final extension reaction at 72 °C for 5 min.

### 3.3. Processing of NGS Data

The Galaxy open-source platform (https://usegalaxy.org/) was used for the conversion of the obtained sequence data (fastq files) to contig files (fasta) and quality files (mothur (version 1.39.5)). A high median quality score was obtained for all investigated data sets.

The contig files were aligned to rRNA databases based on the NCBI cloud using the SILVAngs data analysis service (https://ngs.arb-silva.de/silvangs). It allowed for a detailed community analysis of previously obtained sequencing data [48,49,50]. For all analyses, the pre-set parameter configurations of the SILVAngs database version 138.1 are applied [50].

In many cases, the final obtained sequencing data allow an assignment of taxonomical groups down to the genus level. In other cases, it is only possible to identify higher taxonomical levels as families, orders, classes or phyla. The lowest identified level for each distinguished bacterial type is referenced as the “Operational Taxonomic Unit” (OTU).

In addition to the absolute numbers of reads for each OTU and their percentages or all reads of a sample, a logarithmic value is used here for comparing the compositions of soil bacterial communities. Therefore, the logarithms-related values r is applied, which is defined by the read number N per single OTU and the total number of all reads for each sample N_sum_ as follow:

r = log10(1 + 1000000 × N/N_sum_) (1)

The method of Principal Component Analysis (PCA) was used for a general identification of the relations between the composition patterns of soil bacterial communities of the single samples. The PCA is a statistical method for characterization of large data sets in multidimensional parameter spaces. It allows the reduction of the dimensionality of such parameter spaces and to identify the most important components. It transforms all data from the original multidimensional coordinate system into a new coordinate system with the best fitting for the first coordinate (“First Principal Component” PC1), the second-best fitting for the second coordinate (“Second Principal Component” PC2), and so on. In many cases, the correlation plots of both first coordinates give a sufficient illustration for the clustering of data. Here, the PCA function of MATLAB (R2015b) was used for the calculation of the Principal Components in the abundances of Archaea for the investigated sample set.

## 4. Conclusions

Soil samples from five archaeological excavations have been investigated by 16S r-RNA metagenomics for the presence of *Archaea* in soil bacterial communities. Thereby, considerable differences have been observed. Whereas *Archaea* in one place are nearly negligible, they are present in other samples with more than 10%, in one case more than 40%, of the bacterial population. Samples of the historical tannery area (Jena, Germany) are marked by a high abundance of *Nanoarchaeota* and a considerable abundance of *Asgardarchaeota*. The samples are also marked by high abundances of Ammonia-oxidizing *Crenarchaeota* and by a significant presence of *Zixibacteria* and *Dadabacteria.*

The different excavation places can be distinguished by a Principal Component Analysis (PCA) of archaeal OTUs. This speaks for the fact—despite all differences between specific samples—that a typical pattern of Archaea marks each place. These patterns and the specific compositions of soil bacterial communities in the single samples probably reflect the recent ecological situation and represent the “ecological memory” of former local human impacts dating back centuries or millennia.

In addition to the recognition of differences in archaeal communities from different early human-impacted soils and the consequences of the former human activities on recent local soil bacterial communities, the archaeal strains from these places are also interesting as a potential reservoir for the future search for usable microorganisms in biotechnology. The application of Archaea and their metabolic products is under discussion [51] and soils with different kinds of ancient human impacts should be considered as an interesting special source for new technically usable bacterial strains in the future.

## Data Availability

The data are archived at the authors institution (Techn. Univ. Ilmenau, Germany).

## Supplementary Materials

The following supporting information can be downloaded at: https://www.mdpi.com/article/10.3390/ijms24065519/s1.
