# Supplementary material for: Archaeal and Extremophilic Bacteria from Different Archaeological Excavation Sites
Source: Int J Mol Sci. 2023 Mar 14;24(6):5519. doi: 10.3390/ijms24065519 (PMC10052888; doi:10.3390/ijms24065519)
Supplement: Supplementary file 1 [file ijms-24-05519-s001.zip › ijms-2258453-supplementary.pdf]

Supplementary of

“Archaeal and Extremophilic Bacteria from Different Archaeological Excavation Sites”

(J. Michael Köhler, Linda Ehrhardt and P. Mike Günther)

Table S1: investigated samples and sampling sites

No	Internal Lab No	place of origin	sampling site/situation
01	HB53-1	Kölleda	content of a bronze bowle
02	HB53-2	Kölleda	content of a bronze bowle
03	HB54-1	Großengottern	soil in contact with a bronze hoard
04	HB54-2	Großengottern	soil in contact with a bronze hoard
05	HB57-1	Bennstedt	coal seam
06	HB57-2	Bennstedt	coal seam
07	HB58-1	Bennstedt	replaced sediment
08	HB58-2	Bennstedt	replaced sediment
09	HB59-1	Bennstedt	replaced top soil
10	HB59-2	Bennstedt	replaced top soil
11	HB60-1	Bad Dürrenberg	top soil
12	HB60-2	Bad Dürrenberg	top soil

13	HB61-1	Bad Dürrenberg	red ash deposit
14	HB61-2	Bad Dürrenberg	red ash deposit
15	HB62-1	Bad Dürrenberg	grey ash deposit
16	HB62-2	Bad Dürrenberg	grey ash deposit
17	HB55-1	Jena, Inselplatz	vat environment, depth 1,6 m (Deep1)
18	HB55-2	Jena, Inselplatz	vat environment, depth 1,6 m (Deep1)
19	HB56-1	Jena, Inselplatz	vat environment, depth 2,0 m (Deep2)
20	HB56-2	Jena, Inselplatz	vat environment, depth 2,0 m (Deep2)
21	HB32-1	Jena, Inselplatz	environment of vat
22	HB32-2	Jena, Inselplatz	environment of vat
23	HB33-1	Jena, Inselplatz	interior of a vat
24	HB34-1	Jena, Inselplatz	environment of vat
25	HB34-2	Jena, Inselplatz	environment of vat
26	HB35-3	Jena, Inselplatz	interior of a vat
27	HB35-2	Jena, Inselplatz	interior of a vat
28	HB36-1	Jena, Inselplatz	environment of vat
29	HB36-2	Jena, Inselplatz	environment of vat
30	HB38-1	Jena, Inselplatz	environment of vat
31	HB38-2	Jena, Inselplatz	environment of vat
32	HB39-1	Jena, Inselplatz	interior of a vat
33	HB39-2	Jena, Inselplatz	interior of a vat
34	HB40-1	Jena, Inselplatz	environment of vat
35	HB40-2	Jena, Inselplatz	environment of vat

Table S2: pH-values and electrical conductivity of soils

Internal Lab No	pH	cond [µS/cm]
HB53	9,65	195
HB54	8,68	167
HB57	4,22	115
HB58	4,09	43
HB59	4,05	45
HB60	7,71	1068
HB61	8,24	2100
HB62	8,09	2337
HB55	9,21	78
HB56	9,21	79
HB32	8,09	673
HB33	7,71	1110
HB34	8,37	423
HB35	8,1	512
HB36	8,29	603
HB38	8,6	490
HB39	7,65	586
HB40	8,18	356

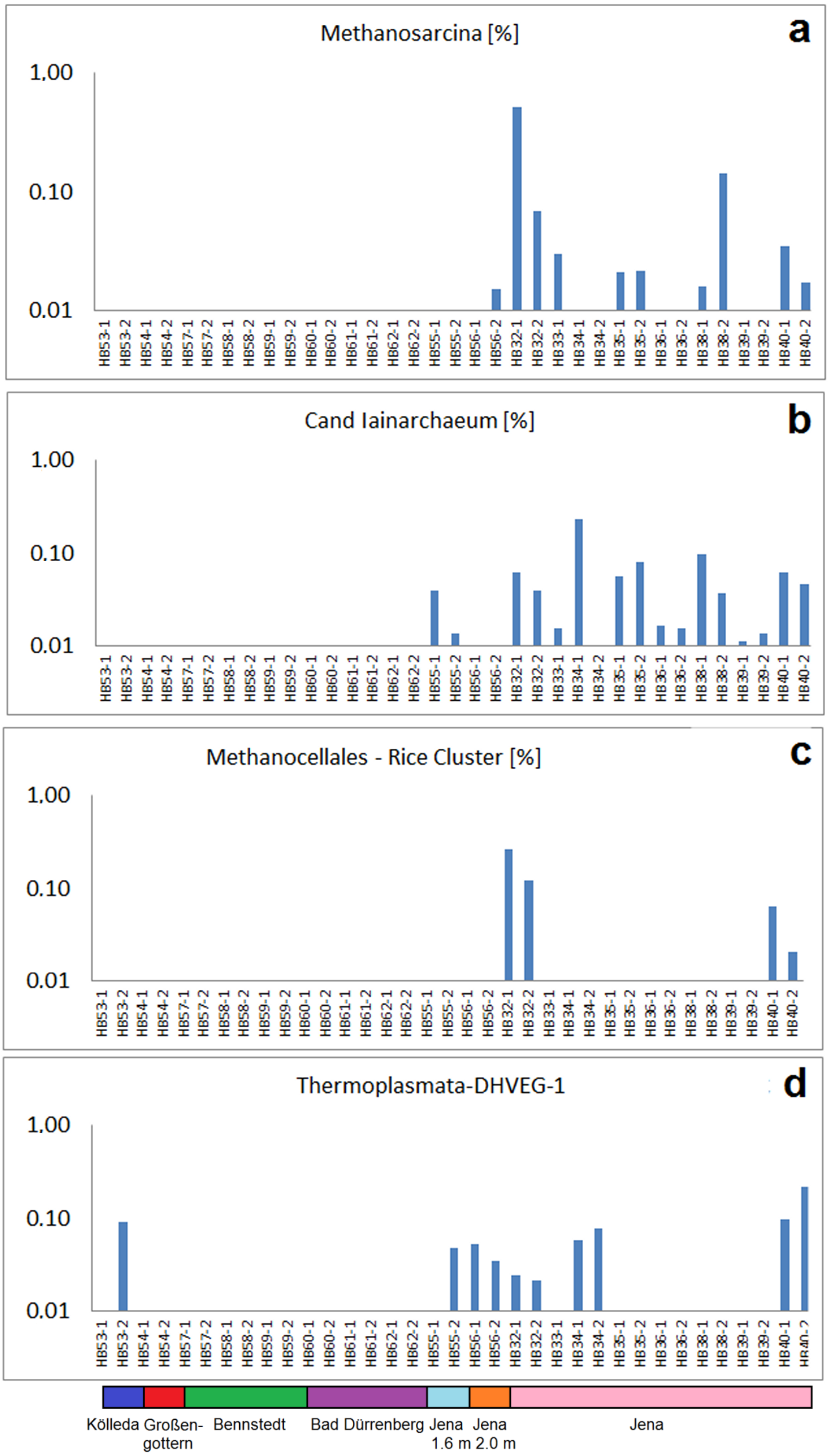

Figure S1 Examples for less abundant OTUs: (a) abundances of *Methanosarcina*, (b) abundances of Cand. *Iainarchaeum*, (c) abundances of *Methanocellales* – Rice Cluster, d) abundances of *Thermoplasmata* group DHVEG-1
